# Prenatal Diagnosis and Postnatal Ultrasound Findings of Cloacal Anomaly: A Case Report

**DOI:** 10.1155/2012/969860

**Published:** 2012-10-04

**Authors:** Lívia Teresa Moreira Rios, Edward Araujo Júnior, Luciano Marcondes Machado Nardozza, Ana Carolina Rabachini Caetano, Antonio Fernandes Moron, Marília da Glória Martins

**Affiliations:** ^1^Mother-Child Unit, Universitary Hospital, Federal University of Maranhão (UFMA), São Luiz, MA, Brazil; ^2^Department of Obstetrics, Federal University of São Paulo (UNIFESP), Rua Carlos Weber, 956 apto. 113 Visage, Alto da Lapa, 05303-000 São Paulo, SP, Brazil

## Abstract

Cloacal malformation is an extremely rare fetal pathological condition that presents as a variety of defects. It predominantly affects females, with prevalence of 1 in 50,000 births. Prenatal ultrasonography on a 20-year-old caucasian woman (G4P1A2) at 33 weeks of pregnancy showed the fetus having a large cystic mass in the lower abdomen with a single septum, bilateral hydronephrosis, ambiguous genitalia, and a single umbilical artery. The pregnancy developed accentuated oligohydramnios, and presence of a fetal brain-sparing effect was diagnosed using arterial Doppler velocimetry. The newborn showed abdominal distension, ambiguous genitalia, and rectal atresia, with a single perineal opening. Pelvic ultrasound done on the first day after delivery revealed the presence of a large retrovesical septated cystic mass of dense content in the fetal abdomen, and bilateral hydronephrosis. Hysterotomy was performed, and 70 mL of dense liquid was drained through an abdominal colostomy. The infant died on the 27th day of life as a result of infectious complications. Prenatal diagnosing of female urogenital anomalies is usually difficult because of their rarity, different types of manifestation, and lack of characteristic ultrasound signs. Presence of a septated cyst with dense content in the fetal abdomen confirms the finding of hydrometrocolpos, thus raising clinical suspicion of a cloacal anomaly.

## 1. Introduction

Cloacal anomalies comprise a rare pathological condition that is observed in female fetuses. They present in various forms depending on the kind of malformation and the gestational age at the time of diagnosis [1, 2]. The observed female prevalence is 1 in 50,000 births for the most common forms and 1 in 250,000 births for the rarest forms, that is, cloacal exstrophy [1, 3].

Cloacal malformation is a condition in which there is a single opening for the genital, urinary, and gastrointestinal tracts. Embryologically, this confluence normally persists only up to the fifth week of pregnancy. Persistence of a cloacal condition is caused by an abnormal development of the urorectal septum [4]. 

Most of the cases that have been described were diagnosed in the third trimester or immediately after birth [3, 4]. The discovery of a megacyst during the first trimester may indicate a diagnosis of cloacal malformation [5]. The main prenatal ultrasound finding is a hypoechoic and septated retrovesical mass, located in the fetal abdomen, but the imaging is mostly inconclusive [6]. In a series of six cases of cloacal malformation that were confirmed during the postnatal period, the main prenatal ultrasound findings were a cystic pelvic mass seen in all six cases, bilateral hydronephrosis seen in all six cases and lack of visible bladder, noted in three cases [7]. In most cases, magnetic resonance imaging is necessary for diagnostic confirmation, since this is capable of showing the communication of the cystic mass with the uterine cavity [6, 8].

We present a rare case of cloacal anomaly, in which the diagnostic suspicion came from obstetric ultrasonography and confirmation came from pelvic ultrasonography on the newborn, in association with the surgical findings.

## 2. Case Presentation

**Figure 1:**
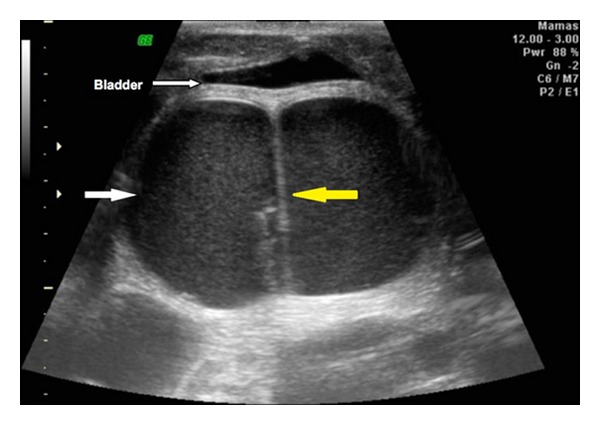
Ultrasound scan in axial plane at the level ofthe fetal pelvic region, shows the bladder brought forward by the cystic mass of dense content (white arrow), at the midsagittal septum (yellow arrow).

**Figure 2:**
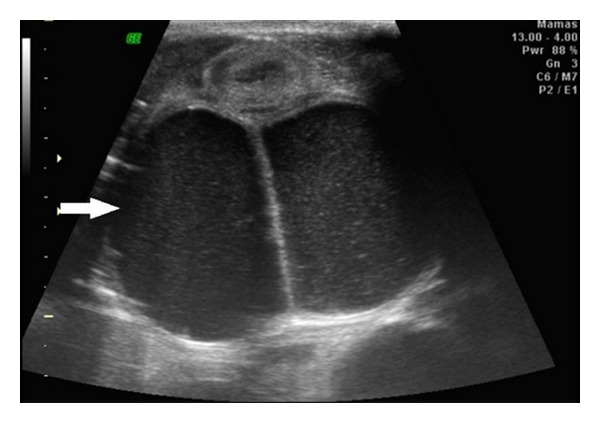
Ultrasound scan after the bladder was completely empty, shows the septated cystic mass (white arrow) occupying the region of the newborn's uterus.

**Figure 3:**
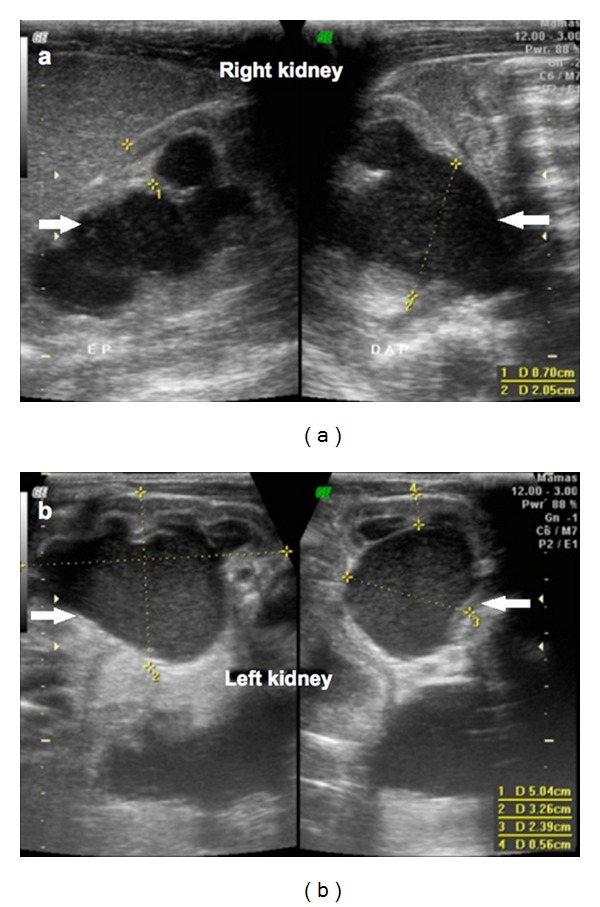
Ultrasound scan in sagittal plane at the level of the kidneys shows bilateral hydronephrosis of dense content (white arrows): (a) in the right kidney; (b) left kidney.

A pregnant 20-year-old white woman (G4P1A2) was referred to our obstetric service with an ultrasound report of a single pregnancy of 33 weeks that had been developing with oligohydramnios and fetal hemodynamic centralization. Morphological assessment on the fetus showed the presence of a large septated cystic mass in the fetal abdomen in the region of the bladder (Figure 1). This mass measured 10.0 × 7.3 × 8.7 cm in size. The fetus showed bilateral hydronephrosis, ambiguous genitalia and a single umbilical artery. The newborn had abdominal distension, simulating a distended bladder, and rectal atresia with a single perineal opening between the labia majora. The newborn was placed in the neonatal intensive care unit, where a diagnostic investigation was conducted. Pelvic ultrasound was done on the first day after delivery. The scan revealed a large retrovesical dense cystic mass with a median septum (Figure 2) in the pelvic region, which reached the level of the umbilical scar. In addition, ultrasound showed bilateral hydronephrosis with dense content, in which the texture was similar to what was observed in the cystic mass (Figure 3). Laparotomy done on the second day of life showed the presence of a double uterus (uterus didelphys) of augmented volume. The uterine content was aspirated using a fine needle, and the result demonstrated the presence of urine. Communication between the uterus and the large intestine was observed. An abdominal colostomy was constructed. The infant died on the 27th day of life due to infectious complications.

## 3. Discussion

Prenatal diagnoses of female urogenital anomalies is usually difficult. These defects are rare, manifest as varying defects and particularly in the late stages of pregnancy lack characteristic ultrasound signs [1–3, 9].

Cloacal malformation (cloacal persistence) is caused by an malformation of the urorectal septum that divides the anorectal canal from the urogenital tract and results in different anomalies such as female hypospadias, double uterus, vesical diverticulum, double vagina, imperforate vagina, or other more complex anomalies [3].

In this case, the cloacal anomaly presented as a double uterus (uterus didelphys), which was identified because of hydrometrocolpos which was revealed by the median septum that divided the two uterine units. The bladder with minimal repletion was identified during ultrasound scan of the newborn by pelvic via; realizing the emptying of bladder, which allowed the understanding that the septate image was the uterus. It is often impossible to distinguish the bladder from the uterus (hydrometrocolpos) on ultrasound images, but this difference is seen accurately through magnetic resonance imaging (MRI) [6, 8]. Hayashi et al. [6] used MRI to confirm a cloacal anomaly in a fetus at 35 weeks. Warne et al. [7] used MRI as a diagnostic complementary method in six cases of cloacal anomaly in fetus between 19 and 33 weeks of gestational age. In our case, MRI was not performed because this equipment was not available at our service, which delayed the diagnostic confirmation until the postnatal period. 

In literature cases of cloacal anomaly have been reported to be associated with fetal ascites. Fetal urine would drain through the Fallopian tube to peritoneal cavity; this process would develop a chemical reaction that would determine the tubal obstruction, hydrometrocolpos, and resolution of ascites [3]. No ascites was observed in our case, probably because of the late diagnosis, by which time the tubal obstruction had already occurred. In our case, we observed that oligohydramnios, which may be caused by severe renal dysfunction, extended hydronephrosis, or difficulty in urine drainage due to a stenosed vagina [10].

Although ultrasonography is able to identify the large abdominal cystic mass, its origin cannot be determined in most cases. The differential diagnosis includes intestinal atresia, ovarian cysts, megabladder-microcolon-intestinal hypoperistalsis syndrome, and obstructive uropathy [3].

In this case, the presence of an abdominal septated cyst in the central region of the fetal pelvis confirmed the finding of uterine duplication with hydrometrocolpos, thus increasing the clinical suspicion of a cloacal anomaly, which was confirmed during the postnatal period.

## References

[1] Meizner I, Levy A, Barnhard Y (1995). Cloacal exstrophy sequence: an exceptional ultrasound diagnosis. *Obstetrics and Gynecology*.

[2] Winderl LM, Silverman RK (1995). Prenatal diagnosis of congenital imperforate hymen. *Obstetrics and Gynecology*.

[3] Odibo AO, Turner GW, Borgida AF, Rodis JF, Campbell WA (1997). Late prenatal ultrasound features of hydrometrocolpos secondary to cloacal anomaly: case reports and review of the literature. *Ultrasound in Obstetrics and Gynecology*.

[4] Adams MC, Ludlow J, Brock JW, Rink CR (1998). Prenatal urinary ascites and persistent cloaca: risk factors for poor drainage of urine or meconium. *Journal of Urology*.

[5] Taipale P, Heinonen K, Kainulainen S, Seuri R, Heinonen S (2004). Cloacal anomaly simulating megalocystis in the first trimester. *Journal of Clinical Ultrasound*.

[6] Hayashi S, Sago H, Kashima K (2005). Prenatal diagnosis of fetal hydrometrocolpos secondary to a cloacal anomaly by magnetic resonance imaging. *Ultrasound in Obstetrics and Gynecology*.

[7] Warne S, Chitty LS, Wilcox DT (2002). Prenatal diagnosis of cloacal anomalies. *British Journal of Urology International*.

[8] Hung YH, Tsai CC, Ou CY, Cheng BH, Yu PC, Hsu TY (2008). Late prenatal diagnosis of hydrometrocolpos secondary to a cloacal anomaly by abdominal ultrasonography with complementary magnetic resonance imaging. *Taiwanese Journal of Obstetrics and Gynecology*.

[9] Respondek-Liberska M, Krason A, Kaczmarek P (1998). Fetal hydrometrocolpos: not only diagnostic but also therapeutic dilemmas. *Ultrasound in Obstetrics and Gynecology*.

[10] Ohno Y, Koyama N, Tsuda M, Arii Y (2000). Antenatal ultrasonographic appearance of a cloacal anomaly. *Obstetrics and Gynecology*.

